# Aortic valve neocuspidization using bovine pericardium for prosthetic valve endocarditis in a hemodialysis patient

**DOI:** 10.1186/s44215-023-00097-4

**Published:** 2023-08-01

**Authors:** Seimei Go, Taiichi Takasaki, Keijiro Katayama, Shinya Takahashi

**Affiliations:** 1 Department of Cardiovascular Surgery, Hiroshima University Hospital, Kasumi 1-2-3, Minamiku Hiroshima-City, Hiroshima, Japan; 2 Department of Cardiovascular Surgery, Fukuyama Cardiovascular Hospital, Fukuyama, Japan

**Keywords:** PVE, AVNeo, Bovine pericardium

## Abstract

**Background:**

Hemodialysis patients are at high risk for prosthetic valve endocarditis (PVE) because of the risk of bacteria entering with each hemodialysis session. To avoid the use of artificial materials as much as possible, we performed aortic valve neocuspidization (AVNeo) using bovine pericardium.

**Case presentation:**

A 67-year-old patient had undergone aortic valve replacement using a bioprosthetic valve 4 months previous. For the past month, he had had PVE caused by *Candida glabrata*. Because the pericardium had already been incised in the previous surgery and there was not enough pericardium left for neocuspidization, we performed AVNeo using bovine pericardium. The previously implanted bioprosthetic valve revealed sticky fungal warts on the leaflet and valve ring, which prevented the movement of the valves. We cut all of the threads holding the valve and removed the bioprosthetic valve. Three valves were cut from the bovine pericardium using a unique template following the method of Ozaki et al. and were fixed to the valve ring with a running suture. The postoperative course was good.

**Conclusions:**

We believe that this treatment is an effective method for PVE because we can avoid the use of artificial materials as much as possible.

## Background

Hemodialysis patients are at high risk for prosthetic valve endocarditis (PVE) because of the risk of bacteria entering with each dialysis session. Although it is common to control the infection with antimicrobial therapy before performing re-valve replacement, reoperation must sometimes be performed in an infected situation because of continued cerebral infarction induced by vegetation dispersal or abscess formation that can dislodge the valve ring and cause rapid valve regurgitation. In such cases, the risk of re-infection after the operation is high, and it is better to avoid the use of artificial materials as much as possible. The clinical efficacy of aortic valve neocuspidization (AVNeo) using glutaraldehyde-treated pericardium, which does not use a prosthetic valve, has been reported [1]. However, obtaining autologous pericardium in re-operative cases is difficult. In this case, we performed AVNeo using bovine pericardium with good results.

## Case presentation

A 67-year-old patient who had undergone hemodialysis for 7 years visited our hospital for severe aortic stenosis. We had performed aortic valve replacement (AVR) using a 23-mm MagnaEase bioprosthetic valve (Edwards LifeSciences, CA, USA) 4 months previous. The patient had had a good postoperative course without serious complications and was discharged 2 weeks later.

**Fig. 1:**
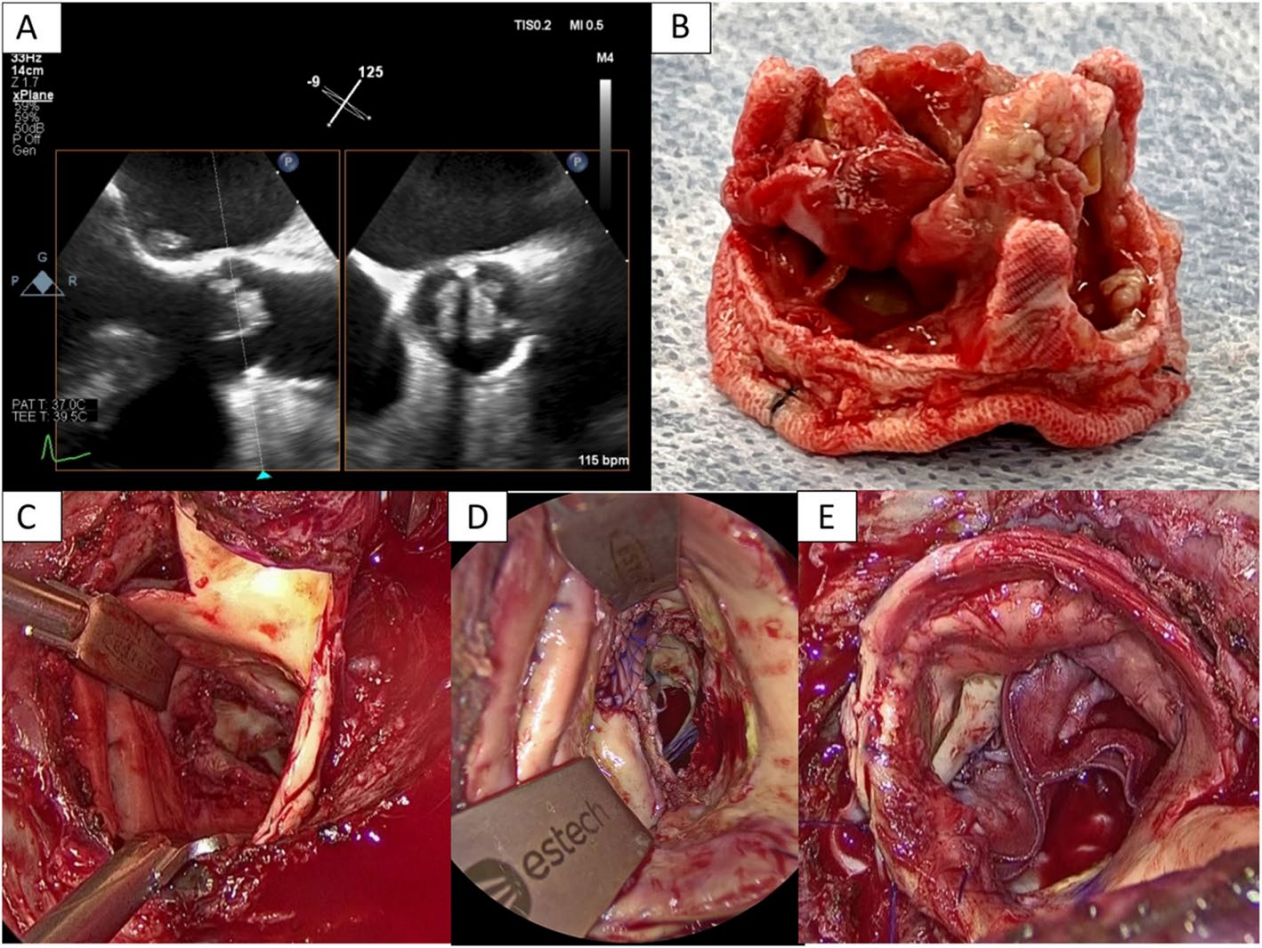
**A** All three valve leaflets were thickened with vegetation, and the mobility of the valve was decreased. **B** The previously implanted bioprosthetic valve revealed sticky fungal warts on the leaflet and valve ring, which prevented the movement of valves. **C** We found an abscess cavity in the valve ring on the NCC side. **D** A 20 mm × 5 mm bovine pericardium patch was first placed at the entrance to the abscess cavity to reinforce the valve ring. **E** The smooth surface of the bovine pericardium was placed on the outflow tract side, and trimmed valves were fixed to the valve ring with a running suture

Over the last month, the patient had had a slight fever and general malaise and gradually began to have pancytopenia. Blood culture revealed *Candida glabrata* infection and echocardiography revealed a 12 mm × 8 mm vegetation adherence to the prosthetic valve. All three valve leaflets were thickened with vegetation, and the mobility of the valve was decreased (Fig. 1A). Head magnetic resonance imaging revealed multiple new cerebral infarcts and showed that the vegetation was still scattered. It was determined that the administration of antifungal drugs did not provide a sufficient therapeutic effect, so we decided to perform a re-operation. Controlling for infection would be difficult with re-valve replacement with bioprosthetic valves as the fungal infection could reattach to the prosthesis easily. AVNeo using autologous pericardium is thought to be more resistant to infection, but the pericardium had already been incised at the previous surgery and there was not enough pericardium left for neocuspidization. Therefore, we decided to perform AVNeo using bovine pericardium.

The chest was incised along the previous wound site, and the sternum was carefully fully sternotomized with a ginkgo-type wound opener. The tissue within the mediastinum was strongly adherent, and the adhesions were carefully detached using an electrocautery and ultrasonic scalpel. After obtaining cardiac arrest with retrograde myocardial protection from the coronary sinus, the ascending aorta was incised. The previously implanted bioprosthetic valve revealed sticky fungal warts on the leaflet and the sewing cuff, which prevented the movement of valves. We cut all of the threads holding the valve and removed the bioprosthetic valve (Fig. 1B). We scraped the valve annulus with a cavitron ultrasonic surgical aspiration to remove the vegetation as much as possible. We also found an abscess cavity in the valve ring on the NCC side (Fig. 1C). We checked the lumen with a sonde and found that it was not in communication with any other lumen.

After cleaning the valve ring area, a 20 mm × 5 mm bovine pericardium patch was first placed at the entrance to the abscess cavity to reinforce the valve ring (Fig. 1D). Three 27 valves were cut from the bovine pericardium using a unique template following the method of Ozaki et al. [1]. The smooth surface of the bovine pericardium was placed on the outflow tract side, and trimmed valves were fixed to the valve ring with a running suture (Fig. 1E).

After confirming that there were no problems with the motion of the reconstructed bovine pericardial Ozaki valve, we removed the cardiopulmonary bypass and finished the operation. The cross-clamp time was 247 min, and the cardiopulmonary bypass time was 310 min. Postoperatively, the patient was treated with antifungal drugs for 6 weeks while undergoing rehabilitation. He was then transferred to a hospital for rehabilitation. Six months postoperatively, the patient is doing well and has had no recurrence of PVE.

## Discussion and conclusions

Fungal infections in dialysis patients are reported to have 9.8 times the morbidity and 1.35 times the mortality compared to the general population [2]. This risk is not unexpected, since hemodialysis patients require frequent direct access to the bloodstream. Although PVE is rare, it is associated with high mortality and morbidity [3]. If a re-operation must be performed in a situation where the infection is uncontrolled, it is better to avoid the use of artificial materials as much as possible. The clinical efficacy of AVNeo using glutaraldehyde-treated pericardium, which does not involve a prosthetic valve, has been reported [1]. However, obtaining autologous pericardium in re-operative cases is difficult. In this case, we used bovine pericardium instead of autologous pericardium for both neocuspidization and for repairing the valve ring.

The neocuspidization valve is reported to be suitable for infectious endocarditis cases. Unlike prosthetic valves, the neocuspidization valve has no stent and preserves the expansion and contraction of the valve ring, making it have better hemodynamics. Dr. Ozaki reported performing AVNeo on 850 patients, using bovine pericardium in 23 of them. In his report, the longest follow-up period was 9 years, with a re-operation-free rate of 100%. Dr. Ozaki recommends the use of bovine pericardium in Japan when autologous pericardium is not available [4]. Dr. Sheng reported 36 cases of neocuspidization using bovine pericardium for aortic valve regurgitation with good mid-term results [5]. Based on these reports, neocuspidization with bovine pericardium is an acceptable surgical technique. Furthermore, Dr. Iida and colleagues reported good results with neocuspidization using bovine pericardium for PVE after AVR, which is similar to our case [6].

Like Dr. Iida’s report, in our case, neocuspidization of the bovine pericardium and administration of antifungal drugs controlled the infection and allowed the patient to heal well and be discharged. We believe that this treatment is an effective method for PVE. However, it has not yet been reported in sufficient numbers, and its long-term prognosis has not yet been reported. Further studies are needed to evaluate the effectiveness of this treatment.

## Data Availability

There are no additional data to disclose.
